# Supplementary material for: 5-Hydroxymethylcytosine in Cell-Free DNA Predicts Immunotherapy Response in Lung Cancer
Source: Cells. 2024 Apr 19;13(8):715. doi: 10.3390/cells13080715 (PMC11049556; doi:10.3390/cells13080715)
Supplement: Supplementary file 1 [file cells-13-00715-s001.zip › Supplementary_Cells_R_F.pdf]

5-hydroxymethylcytosine in cell-free DNA predicts immunotherapy response in lung cancer

Jianming Shao, Yitian Xu, Randall J. Olsen, Saro Kasparian, Kai Sun, Sunil Mathur, Jun Zhang, Chuan He, Shu-Hsia Chen, Eric H. Bernicker, Zejuan Li

Contents

Table S1. Patient demographics and baseline characteristics. ……………………………………2

Table S2. List of genes associated with immune checkpoint inhibitor treatment response. ……………………………………………………………………………… Attachment

Table S3. Wp-scores of lung cancer samples treated with immunotherapy. ……….. Attachment

Table S4. Multivariate Cox regression analysis in patients with PD-L1 expression. …..……….. 3

Table S5. Therapeutic response to immune checkpoint inhibitors predicted by tumor PD-L1 expression. ……………………………………………………………………………………… 3

Table S6. Prediction accuracy by the 5hmC signature and tumor PD-L1 expression. ……….... 4

Figure S1. Study design for cell-free DNA 5hmC predictive model for lung cancer immunotherapy. ………………………………..……………………..……………..……...….... 5

Figure S2. Prediction of overall survival by a 5hmC predictive signature in lung cancer patients receiving immune checkpoint inhibitor treatment. …………………………………………………6

Figure S3. Immune checkpoint inhibitor treatment response predicted by tumor PD-L1 expression. ………………………………………………………..………………………..…...... 7

Figure S4. Treatment response predicted by the 5hmC predictive signature in lung cancer patients not receiving immune checkpoint inhibitors. ……………………………………......... 8

Table S1. Patient demographics and baseline characteristics.

Characteristics	All patients (N=83)	Training (n=24)	Validation (n=16)	Test (n=18)	Non-ICI group (n=27)
**Age, median (range), y**	69 (48-92)	70 (50-92)	72 (48-90)	67 (53-83)	69 (51-89)
**Female, No. (%)**	45 (54.2)	9 (37.5)	9 (56.2)	8 (44.4)	20 (74.1)
**Race, No. (%)**					
**Asian**	4 (4.8)	0	0	1 (5.6)	3 (11.1)
**Black**	18 (21.7)	6 (25.0)	4 (25.0)	1 (5.6)	7 (25.9)
**Caucasian**	60 (72.3)	18 (75.0)	12 (75.0)	16 (88.9)	16 (59.3)
**Unavailable**	1 (1.2)	0	0	0	1 (3.7)
**ECOG PS, No. (%)**					
**0**	6 (7.2)	1 (4.2)	2 (12.5)	2 (11.1)	1 (3.7)
**1**	62 (74.7)	19 (79.2)	13 (81.2)	16 (88.9)	16 (59.3)
**2**	1 (1.2)	1 (4.2)	0	0	0
**3**	2 (2.4)	1 (4.2)	0	0	1 (3.7)
**4**	3 (3.6)	1 (4.2)	0	0	2 (7.4)
**Unavailable**	9 (10.8)	1 (4.2)	1 (6.2)	0	7 (25.9)
**Smoking status, No. (%)**					
**Current**	13 (15.7)	3 (12.5)	2 (12.5)	3 (16.7)	5 (18.5)
**Former**	57 (68.7)	20 (83.3)	13 (81.2)	15 (83.3)	11 (40.7)
**Never**	13 (15.7)	1 (4.2)	1 (6.2)	0	11 (40.7)
**Histology, No. (%)**					
**NSCLC**					
**Adenocarcinoma**	59 (71.1)	18 (75.0)	8 (50.0)	13 (72.2)	22 (81.5)
**Squamous**	19 (22.9)	6 (25.0)	6 (37.5)	4 (22.2)	3 (11.1)
**Other subtypes**	3 (3.6)	0	2 (12.5)	1 (5.6)	0
**SCLC**	2 (2.4)	0	0	0	2 (7.4)
**Disease stage, No. (%)**					
**III**	12 (14.5)	2 (8.3)	5 (31.2)	0	5 (18.5)
**IV**	71 (85.5)	22 (91.7)	11 (68.8)	18 (100.0)	22 (81.5)
**PD-L1 expression, No. (%)**					
**<1%**	42 (50.6)	14 (58.3)	9 (56.2)	10 (55.6)	11 (40.7)
**≥1%**	32 (38.6)	9 (37.5)	5 (31.2)	8 (44.4)	10 (37.0)
**Unavailable**	9 (10.8)	1 (4.2)	2 (12.5)	0	6 (22.2)
**PFS, median (range), mo**	6.6 (0.2-63.1)	7.0 (1.4-34.1)	6.3 (0.5-42.4)	8.7 (1.8-63.1)	5.4 (0.2-56.2)
**OS, median (range), mo**	12.4 (0.2-63.1)	14.1 (1.4-40.5)	9.8 (2.5-42.4)	17.8 (2.1-63.1)	9.1 (0.2-56.2)

ECOG PS, Eastern Cooperative Oncology Group performance status score; NSCLC, non-small cell lung cancer; SCLC, small cell lung cancer; ICI, immune checkpoint inhibitor; PFS, progression-free survival; OS, overall survival.

Table S4. Multivariate Cox regression analysis in patients treated with immune checkpoint inhibitors.

	HR (95% CI)	*P*
Wp-score (Low vs High)	0.11 (0.04-0.29)	9.30×10^{-6}
Age (≥ 60 y vs <60 y)	0.69 (0.26-1.90)	0.47
Sex (Female vs Male)	0.57 (0.27-1.20)	0.13
Race (Caucasian vs Black)	0.88 (0.39-2.00)	0.76
Smoker (Current vs Former)	2.60 (1.00-6.20)	0.04
PD-L1 TPS (≥ 1% vs <1%)	0.73 (0.36-1.50)	0.38

Progression-free survival multivariate Cox regression analysis were performed in 51 patients treated with immune checkpoint inhibitors, considering wp-score (low vs high), age (≥ 60 vs < 60-year-old), sex (female vs male), race (Caucasian vs Black), smoking status (current vs former), and tumor PD-L1 TPS (≥ 1% vs <1%). Wp-score, weighted-predictive score. TPS, PD-L1 tumor proportion score.HR, hazard ratio. CI, confidence interval.

Table S5. Therapeutic response to immune checkpoint inhibitors predicted by tumor PD-L1 expression.

Non-STOMP

	Responders (No.)	Non-responders (No.)	Objective response rate (95% CI)
PD-L1 TPS ≥ 1%	7	6	53.9% (25.1%-80.8%)
PD-L1 TPS < 1%	11	8	57.9% (33.5%-79.8%)

STOMP clinical trial

	Responders (No.)	Non-responders (No.)	Objective response rate (95% CI)
PD-L1 TPS ≥ 1%	3	5	37.5% (8.5%-75.5%)
PD-L1 TPS < 1%	5	5	50.0% (18.7%-81.3%)

Responders: patients with complete or partial response to immune checkpoint inhibitor treatment who did not progress within six months after the treatment. Non-responders: patients with progression or stable disease or with response but progressed within six months after ICI treatment. TPS, PD-L1 tumor proportion score.

Table S6. Prediction accuracy by the 5hmC signature and tumor PD-L1 expression.

Non-STOMP

	**Consistent (No.)**	**Inconsistent (No.)**	**Overall accuracy (95% CI)**
Wp-score	27	5	84.4% (67.2%-94.7%)
PD-L1	15	17	46.9% (29.1%-65.3%)

STOMP clinical trial

	**Consistent (No.)**	**Inconsistent (No.)**	**Overall accuracy (95% CI)**
Wp-score	16	2	88.9% (65.3%-98.6%)
PD-L1	8	10	44.4% (21.5%-69.2%)

Consistent indicates the prediction is consistent with clinical response status. Inconsistent indicates the prediction is not consistent with clinical response status. CI, confidence interval.

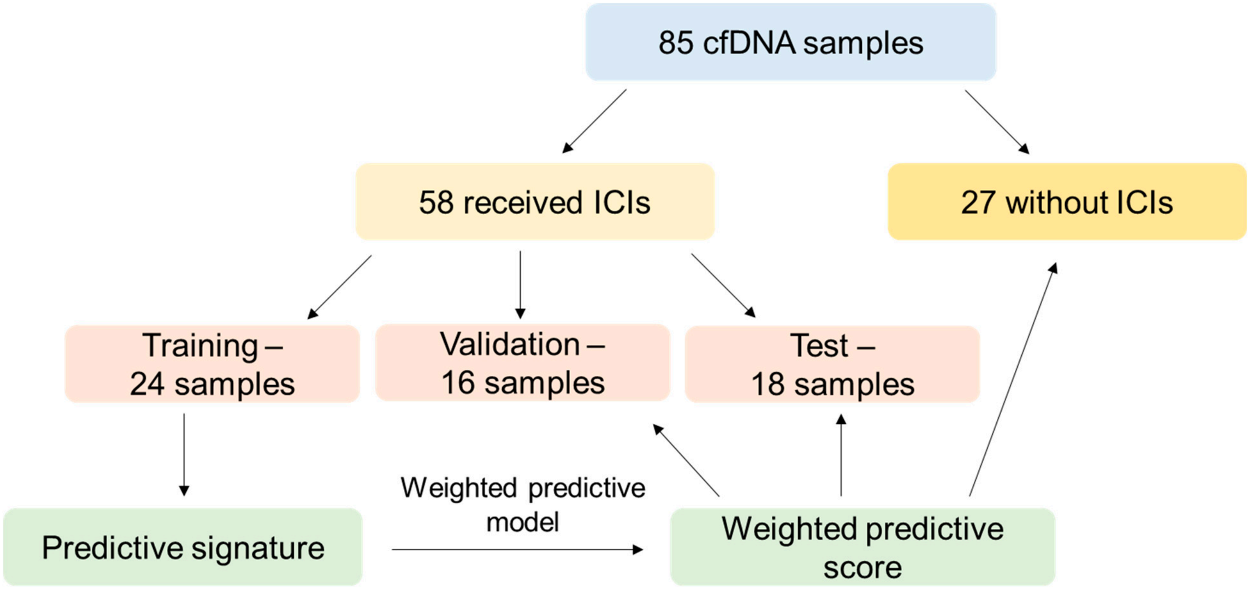

Figure S1. Study design for cell-free DNA 5hmC predictive model for lung cancer immunotherapy.

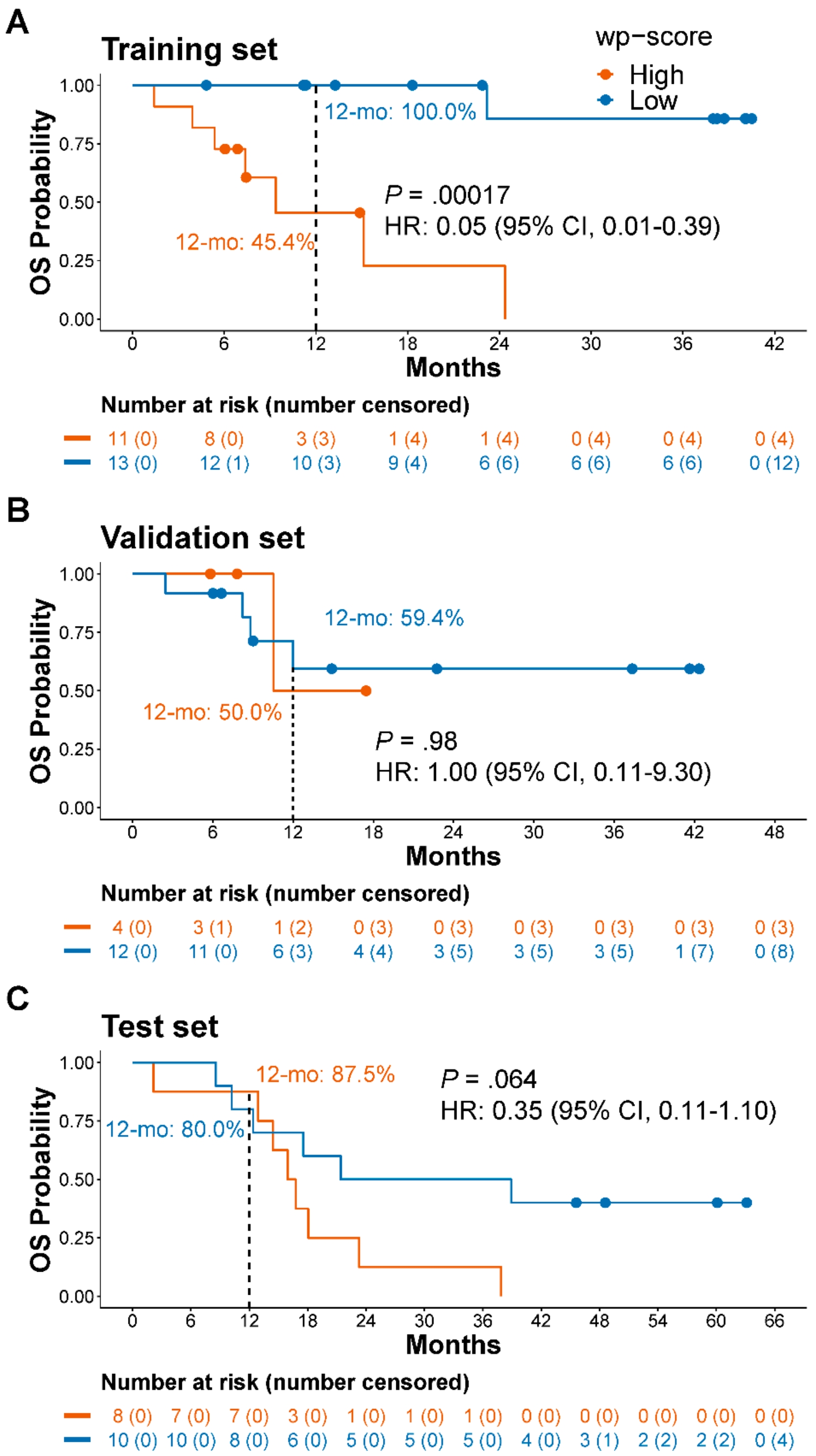

Figure S2. Prediction of overall survival by a 5hmC predictive signature in lung cancer patients receiving immune checkpoint inhibitor treatment. (A, B, C) Kaplan-Meier analysis of overall survival (OS) based on weighted predictive (wp)-scores in the training set (A), the validation set (B), and the test set (C). 12-mo, estimated OS in 12 months. Dots on the survival curve indicate that a patient was censored. HR, hazard ratio. CI, confidence interval.

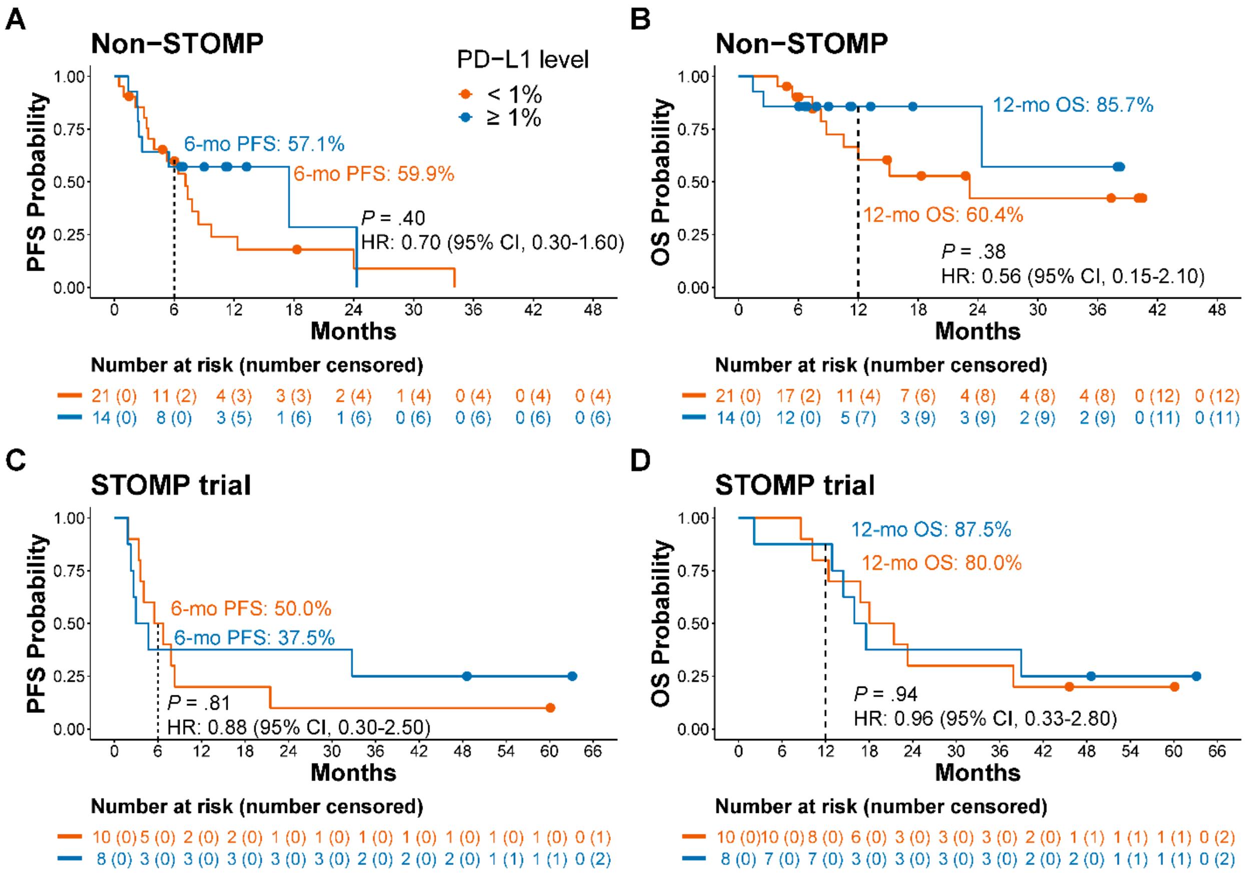

Figure S3. Immune checkpoint inhibitor treatment response predicted by tumor PD-L1 expression. Kaplan-Meier analysis of progression-free survival (PFS, A) and overall survival (OS, B) in non-STOMP patients treated with immune checkpoint inhibitors based on tumor PD-L1 expression. Kaplan-Meier analysis of PFS (C) and OS (D) in STOMP clinical trial patients treated with immune checkpoint inhibitors based on tumor PD-L1 expression. HR, hazard ratio. CI, confidence interval.

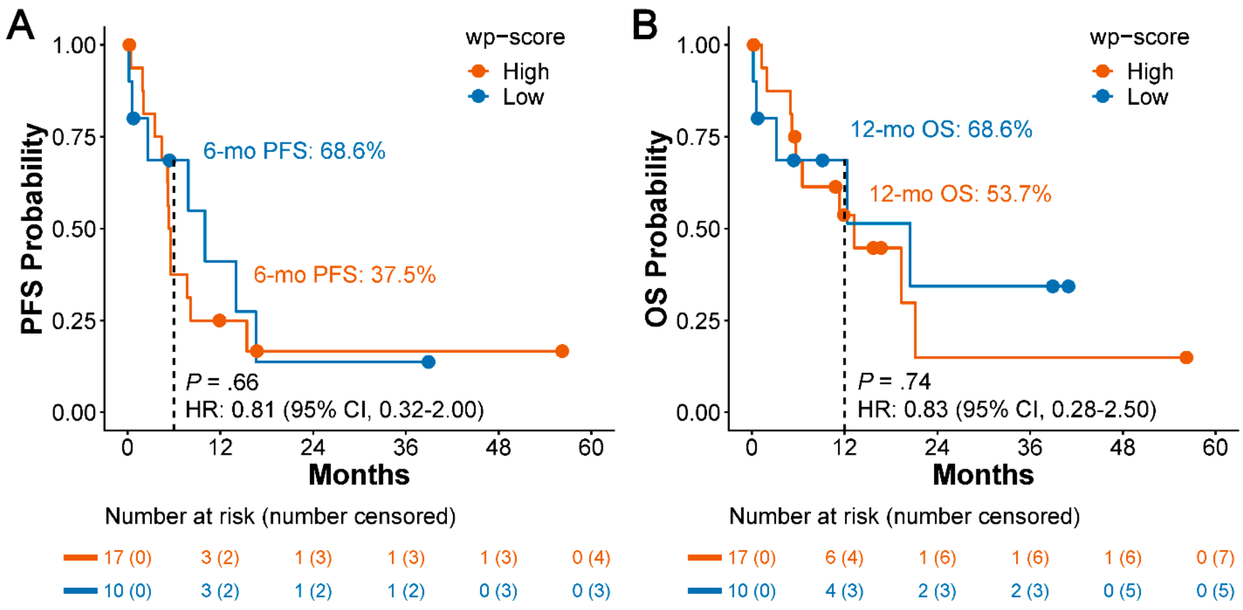

Figure S4. Treatment response predicted by the 5hmC predictive signature in lung cancer patients not receiving immune checkpoint inhibitors. Kaplan-Meier analysis of progression-free survival (PFS, A) and overall survival (OS, B) in patients who did not receive immune checkpoint inhibitor treatment based on weighted prediction (wp)-scores.
